# Cancer predisposing BARD1 mutations in breast-ovarian cancer families

**DOI:** 10.1186/1897-4287-10-S4-A10

**Published:** 2012-12-10

**Authors:** Magdalena Ratajska, Magdalena Matusiak, Izabela Brożek, Maciej Stukan, Marcin Śniadecki, Jarosław Dębniak, Dariusz Wydra, Janusz Limon

**Affiliations:** 1 Department of Biology and Genetics, Medical University of Gdansk, Gdansk, Poland; 2 Department of Gynecology, Gynecologic Oncology and Gynecologic Endocrinology, Medical University in Gdańsk, Poland

## 

BARD1 was identified as a protein interacting with BRCA1 - the heterodimer formed by BRCA1 and BARD1 acts in DNA repair, RNA processing, transcription and cell cycle regulation. BARD1 has also BRCA1-independent functions like mediating p53-dependent apoptosis. Additionally, BARD1 mRNA isoforms were found to be highly expressed in most human gynecological cancers.

We report 17 different *BARD1* variants, four of which were suspected to be pathogenic, including a novel substitution (c.1361C>T) leading to amino acid change in highly conserved ankirin domain motif, a splice mutation (c.1315-2A/G) resulting in exon 5 skipping and a silent change (c.1977A/G) which alters several ESE motifs in exon 10, and results in a transcript lacking exons 2-9. Finally we identified two unrelated patients carrying truncating nonsense mutation in exon 8 (c.1690C>T).

Our findings suggest that *BARD1* mutations may be regarded as cancer risk alleles and warrant further investigation to determine their actual contribution to non-BRCA1/2 breast and ovarian cancer families.

